# Analysis By Deep Sequencing of Discontinued Neurotropic Yellow Fever Vaccine Strains

**DOI:** 10.1038/s41598-018-31085-2

**Published:** 2018-09-07

**Authors:** Andrew S. Beck, Thomas G. Wood, Steven G. Widen, Jill K Thompson, Alan D. T. Barrett

**Affiliations:** 1 Department of Pathology, Galveston, TX 77555 USA; 2 Molecular Genomics Core Facility, Galveston, TX 77555 USA; 3 Sealy Institute for Vaccine Sciences, and World Health Organization Collaborating Center for Vaccine Research, Evaluation and Training on Emerging Infectious Diseases, Galveston, TX 77555 USA; 4 University of Texas Medical Branch, Galveston, TX 77555 USA

## Abstract

Deep sequencing of live-attenuated viral vaccines has focused on vaccines in current use. Here we report characterization of a discontinued live yellow fever (YF) vaccine associated with severe adverse events. The French neurotropic vaccine (FNV) strain of YF virus was derived empirically in 1930 by 260 passages of wild-type French viscerotropic virus (FVV) in mouse brain. The vaccine was administered extensively in French-speaking Africa until discontinuation in 1982, due to high rates of post-vaccination encephalitis in children. Using rare archive strains of FNV, viral RNAs were sequenced and analyzed by massively parallel, *in silico* methods. Diversity and specific population structures were compared in reference to the wild-type parental strain FVV, and between the vaccine strains themselves. Lower abundance of polymorphism content was observed for FNV strains relative to FVV. Although the vaccines were of lower diversity than FVV, heterogeneity between the vaccines was observed. Reversion to wild-type identity was variably observed in the FNV strains. Specific population structures were recovered from vaccines with neurotropic properties; loss of neurotropism in mice was associated with abundance of wild-type RNA populations. The analysis provides novel sequence evidence that FNV is genetically unstable, and that adaptation of FNV contributed to the neurotropic adverse phenotype.

## Introduction

Phenotypic adaptation of viruses arising from serial culture in cell culture or animals is ubiquitous, and constitutes the fundamental property of empirical derivation for live- attenuated vaccines. Of particular significance to the study of live-attenuated vaccines are those administered to prevent infection by yellow fever virus (YFV), which are among the oldest live attenuated vaccines. YFV is the prototype member of the family *Flaviviridae* with a positive-sense RNA genome of 10.8 kb, which is translated as a single open reading frame upon host infection^1^. Wild-type disease manifestations of YFV infection are viscerotropic, referring to tropism of the virus to the liver and resultant hepatocyte injury, associated with characteristic jaundice and hemorrhage in advanced cases^2^.

YFV was a considerable obstruction to economic development until the empiric derivation of two live-attenuated vaccine strains in the early twentieth century. The vaccines arose from separately isolated parental strains, and distinct attenuating subculture methods. The vaccine strain 17D was developed by passage of wild-type strain Asibi in mouse and chicken tissue in 1936 by the Rockefeller Foundation (New York, USA); derivatives of this strain remain in production today and are administered by the subcutaneous route^3^. Concurrent efforts by the Institut Pasteur (Dakar, Senegal) produced the French neurotropic vaccine (FNV), which was administered extensively in French-speaking Africa starting in 1940. The vaccine was produced from a wild-type parental strain known as the French viscerotropic virus (FVV), isolated in 1927 from the Lebanese patient Françoise Mayali. FVV was serially passaged 128 times in mouse brain; the resultant strain was attenuated with respect to both viscerotropism and vector competence; however, neurotropic properties were enhanced^4–6^. FNV was administered by scarification from a reconstitution of infected and desiccated mouse brain, and possessed desirable properties of thermostability and high immunogenicity^6,7^. Commercial lots were administered between passages 250 through 260, relative to the parental strain^6^. Deployment of the FNV is credited as a key intervention in the suppression of YF disease in French West Africa between 1940 and 1954, with over 84 million doses distributed during that time^6,8^. However, FNV was noted for unacceptable rates of post-vaccinal encephalitis in children, constituting a rationale for restricting administration of the vaccine to those above 10 years of age in 1960, and later discontinuance of the vaccine in 1982 due to the availability of the safer 17D strain (which is routinely given to those aged 9 months and older^9^.

Mechanisms of neutrotropism for YFV vaccines are poorly understood, and information on the FNV is particularly obscure. Reference material for FNV is of very limited availability, and passage histories are not generally accessible. YFV strains are variably neurotropic when directly introduced to central nervous tissues of mice, a property that is exploited in lethality and serological protection assays^10–13^. Both rhesus and cynomolgous macaques are in some cases susceptible to neurotropic disease when challenged intracerebrally with YFV vaccine strains, while wild-type strains are not neurotropic and cause viscerotropic disease even when introduced to the brain; this property forms the basis of the current World Health Organization standard assessment of neurotropism for YFV 17D vaccine seed lots^14^.

There are multiple, competing hypotheses available to explain the adverse neurotropic events attributed to administration of FNV. First, it is plausible that the adaptation of the virus to mouse brain conferred an enhanced capacity of the vaccine to infect mammalian central nervous tissues. Second, it is possible that FNV in some cases accumulates wild-type sequence content once administered. We have previously shown that the 17D vaccine genome is genetically highly stable, accumulating nucleotide substitutions mutations very infrequently^15^, whereas the FNV strain genome is highly unstable under laboratory conditions. This instability of FNV was previously observed after few cell culture passages, with concomitant alterations to mouse neurotropism, appearance of wild-type genotypes, and greater consensus sequence variation than the 17D vaccine^16^. Only one example of FNV has been fully sequenced in reference to the parental wild-type FVV strain, revealing 77 nucleotide changes and 33 amino acid substitutions^17^. Other FNV strains were partially sequenced in the study of Wang *et al*., which were unequally neurotropic in mice and monkeys. In comparison, studies of 17D vaccine produced from different substrains (17D-204, 17DD and 17D-213) with different passage histories all show very limited genetic variation^18^. Thus, the co-observation of variable neurotropic phenotypes in mice and monkeys with sequence instability in FNV strains leads to a reasonable hypothesis that incompletely fixed attenuation determinants are present in the population structures of these vaccines. This study investigates these population structures using *in silico* techniques.

## Results

Four examples of the FNV (FNV-IP, FNV-FC, FNV-Yale and FNV-NT), 17D-204 vaccine and wild-type parental strains Asibi and FVV were all subjected to next generation sequencing (Table 1). All viruses were passaged once in Vero cells prior to sequencing, save 17D-204 which was amplified directly from reconstituted vaccine.

**Table 1:** Properties and institutional sourcing for virus strains sequenced in the study.

Strain	Source	Neurovirulence (Previous)		
		Monkey i.c. AST(d)	Mouse i.c. AST (d)	Mouse i.n. LD50 (PFU)
FVV	WRCEVA*			
FNV-IP	Institut Pasteur, Paris	6	5.8	4.1
FNV-Yale	Yale Arbovirus Collection	NT	5.8	4.1
FNV-FC	CDC Fort Collins, CO	9	11.1	4.1
FNV-NT	Porton Down (Public Health England)	>30	5.8	>5.6
Asibi	WRCEVA			
17D-204	Sanofi-Pasteur			

*In vivo* neurovirulence studies were previously reported for these strains, and showed that the collection strains of FNV are unequally neurotropic^17,32^. Note: FVV was previously assayed in suckling mice and is uniformly lethal at 21dpi using a 100PFU intraperitoneal dose^32^. *World Reference Center for Emerging Viruses and Arboviruses (Galveston, TX USA).

### Analysis of Consensus Sequences

**Figure 1:**
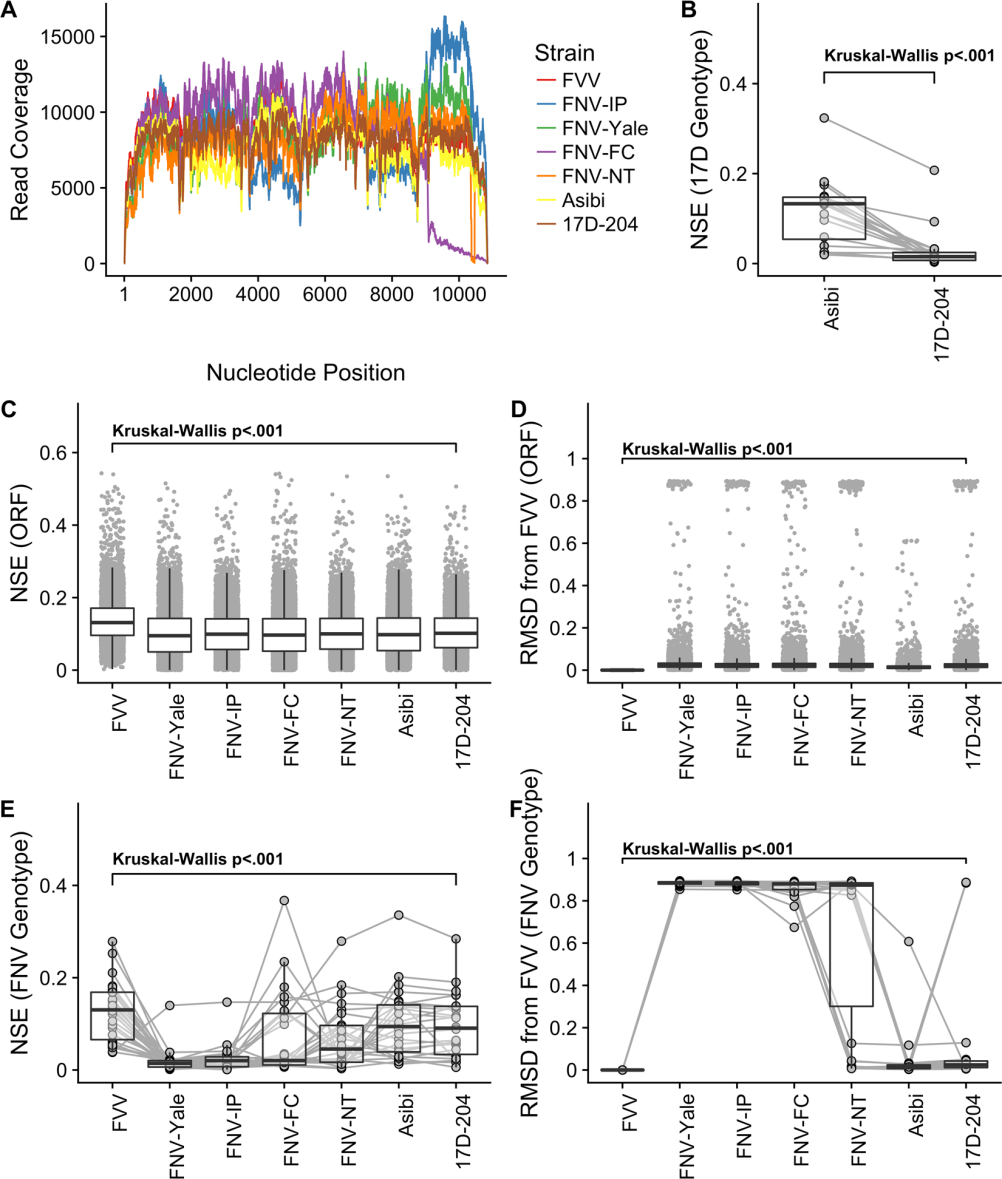
Diversity (normalized Shannon’s entropy - NSE) and genetic distance (Root-mean squared distance - RMSD) from FVV were measured for all nucleotide positions in the single, open reading frame (ORF) of the YFV genomes. Vaccine strains were of lower diversity than wild-type parental strains, especially when exclusively considering nucleotide positions encoding amino acid substitutions that define the vaccine genotypes of FNV or 17D-204. All FNV vaccine strains were of lower diversity than the parental strain FVV, and likewise were of greater genetic distance from FVV. FNV-IP and FNV-Yale were of lowest diversity, whereas FNV-FC and FNC-NT were not stably fixed at the putative vaccine genotype (Table 2). (**A**) Sequencing read coverage for all viruses sequenced in the study, after random downsampling to normalize. **(B)** Paired comparison of NSE for Asibi and 17D-204, for the 20 nucleotide positions that encode amino acid substitutions observed in 17D vaccine substrains. This is provided as a method control to replicate the low-diversity pattern shown previously for 17D-204. **(C)** NSE for all nucleotide positions in viral ORFs. **(D)** RMSD relative to FVV for all nucleotide positions in viral ORFs. **(E)** NSE for 26 nucleotide positions encoding amino acid substitutions relative to FVV in at least 3 of 4 FNV strains. **(F)** RMSD for 26 nucleotide positions depicted in subfigure **E**.

Read alignments were analyzed to identify the consensus sequences for each virus. All alignments were of very deep coverage (Fig. 1A), and so the recovery of consensus (Table 2) and variant information is provided at high confidence.

**Table 2:** Consensus alignment of FNV strains against the wild-type parent FVV.

Gene	Nucleotide Position	Codon	FVV	Asibi	FNV-IP	FNV-Yale	FNV-FC	FNV-NT
C	275	53	F	—	—	—	—	L
	**357**	**80**	**V**	**—**	**A**	**A**	**A**	**A**
	**386**	**90**	**S**	**—**	**G**	**G**	**G**	**G**
prM	**554**	**25**	**V**	**—**	**M**	**M**	**M**	**M**
	596	39	Y	—	—	—	—	H
M	854*	**36**	**L**	**—**	**F**	**F**	**F**	**F**
	992	7	T	—	A	A	A	**
	**1134**	**54**	**A**	**—**	**V**	**V**	**V**	**V**
	1392	140	R	—	—	—	—	K
	1395	141	A	—	—	—	—	V
	1424	151	N	—	—	—	—	D
	1432	153	N	—	K	K	K	**
E	1437	155	D	—	—	—	—	A
	1440	156	I	—	—	—	—	T
	1572* ^**,+**^	**200**	**T**	**K**	**K**	**K**	**K**	**K**
	**1718**	**249**	**N**	**—**	**D**	**D**	**D**	**D**
	1965*	**331**	**K**	**—**	**R**	**R**	**R**	**R**
	2193	407	A	—	—	—	—	V
	2344	457	M	—	I	I	I	**
NS1	3234	261	K	—	—	—	—	R
	3469	339	K	—	—	—	—	N
NS2A	**3752**	**82**	**I**	**—**	**V**	**V**	**V**	**V**
	**3821**	**105**	**T**	**—**	**A**	**A**	**A**	**A**
NS2B	4212	11	A	—	—	—	—	V
	**4358**	**60**	**S**	**—**	**A**	**A**	**A**	**A**
	4464	95	D	—	—	—	—	G
	4550	124	H	—	—	—	—	Y
NS3	4607	13	I	—	V	V	V	**
	4754	62	R	—	—	—	—	W
	4878	103	V	—	—	—	—	A
	**5145**	**192**	**M**	**—**	**K**	**K**	**K**	**K**
	5408	280	I	V	—	—	—	—
NS4A	6459	7	V	—	—	—	—	A
	**6527**	**29**	**F**	**—**	**L**	**L**	**L**	**L**
	6602	54	M	—	—	—	—	L
	6761	107	M	—	—	—	—	L
	**6843**	**135**	**Y**	**—**	**F**	**F**	**F**	**F**
	6875	145	V	—	—	—	—	L
NS4B	6942	19	L	—	S	S	S	**
	6953	22	S	—	—	—	—	G
	7171*	**95**	**I**	**—**	**M**	**M**	**M**	**M**
	**7178**	**97**	**V**	**—**	**I**	**I**	**I**	**I**
	**7380**	**165**	**A**	**—**	**V**	**V**	**V**	**
NS5	7641	2	R	—	T	T	T	**
	7642	2	R	—	—	—	—	S
	7694	20	D	—	—	—	—	N
	8030	132	I	—	—	—	—	V
	8090	152	S	—	—	—	—	P
	**8409**	**258**	**I**	**—**	**T**	**T**	**T**	**T**
	**8640**	**335**	**R**	**—**	**K**	**K**	**K**	**K**
	8928	431	K	—	—	—	—	R
	8939	435	L	—	—	—	—	M
	**9227**	**531**	**F**	**—**	**L**	**L**	**L**	**L**
	9605	657	N	—	—	—	—	D
	9615	660	K	—	—	R	—	R
	10120	828	M	—	—	—	—	I
	10268	878	I	—	—	V	—	V
	10338	901	P	—	L	—	L	—
3′UTR	10357	—	—	—	—	U	—	U
	10358	—	—	—	—	G	—	G
	10363	—	—	—	—	—	—	U
	10367	—	—	—	C	—	C	—
	10404	—	—	—	—	A	—	—
	10418	—	—	—	C	—	C	—
	10550	—	—	—	C	—	C	—
	10554	—	—	—	—	—	—	U
	10798	—	—	—	—	G	—	G
	10800	—	—	G	—	—	—	—

All sites observing amino acid substitutions are shown, with the 19 sites conserved between all vaccine strains highlighted in bold. Several of the sites are revertant to wild-type identity in FNV-NT only, and thus are presumed to be lost on passage; these 26 amino acid substitutions are currently reported as the consensus FNV genotype. *Substitution is observed in the attenuating passage of 17D from Asibi. **Consensus-level revertant of FNV strain to wild-type. ^+^Substitution was observed across Asibi and all FNV strains.

#### Putative Consensus Genotype of FNV

For FNV-IP, 60 nucleotide differences were observed compared to the wild-type parent FVV, including 27 amino acid substitutions. For FNV-Yale, 63 nucleotide differences were observed, including 28 amino acid substitutions. For FNV-FC, 60 nucleotide differences were observed, including 27 amino acid substitutions. For FNV-NT, 122 nucleotide differences were observed, including 49 amino acid substitutions. Overall, 19 common amino acid substitutions are conserved between all four FNV strains when compared to wild-type FVV (Table 2), however 26 of these substitutions are conserved by the FNV strains at 75 percent. Since further data in this study reports sequence instability in some of the vaccine strains, these 26 sites were presumed to describe a consensus genotype of FNV relative to FVV and were analyzed as such.

#### Comparison of FVV to Asibi

For comparison of the wild-type FVV to the Asibi strain, 11 nucleotide differences were observed, with 2 amino acid substitutions located at FVV positions 1572 (E-K200T) and 5408 (NS3-V279I), and 1 nucleotide difference in the 3′ untranslated region at position 10800 (A-G). The close sequence relationship of FVV and Asibi was expected as both were obtained in the same year, presumably from the same West African YF epidemic. For 17D-204 (YF-Vax®), 64 nucleotide differences were observed from wild-type strain Asibi, of which 31 were amino acid substitutions. We have previously published a comparison of YF-Vax® to Asibi and the results obtained here were the same as for the consensus sequences previously reported^15^.

### Diversity of Strains And Genetic Distance from Wild-Type

All statistical tests are performed along the entire set of seven viruses that were analyzed in the study: FNV (x4), FVV, Asibi, 17D-204. Root-mean square distance (RMSD) is reported using FVV as a control value. Adjusted p-values are reported.

#### Open Reading Frames

Normalized Shannon’s entropy (NSE) was calculated along open reading frames of all strains sequenced, and is shown in Table 3A and Fig. 1C. A single-factor Kruskal-Wallis test showed a significant difference for NSE within the aggregate group (X^2^ = 2773.51, df = 6, p < 0.001). A pairwise Wilcoxon sign-rank test revealed significant differences in NSE between all pairs considered (n = 7), except for the pairs Asibi/FNV-IP (p = 0.36), Asibi/FNV-NT (p = 0.11), FNV-FC/FNV-Yale (p = 0.53), and FNV-IP/FNV-NT (p = 0.5). Likewise, RMSD was calculated along open reading frames of all strains sequenced, and is shown in Table 3A and Fig. 1D. Single-factor Kruskal-Wallis test showed a significant difference of RMSD between the strains (X^2^ = 29970, df = 6, p < 0.001). A pairwise Wilcoxon sign-rank test revealed significant differences in RMSD between all pairs considered (n = 7), except for the pairs Asibi/FNV-IP (p = 0.36), Asibi/FNV-NT (p = 0.11), FNV-FC/FNV-Yale (p = 0.53), and FNV-IP/FNV-NT (p = 0.5).

**Table 3:** Diversity and genetic distance statistics for the viruses analyzed in the study.

	NSE			RMSD from FVV		
	Mean	Median	s.d.	Mean	Median	s.d.
**A**	**Open Reading Frame (n = 10236)**					
FVV	0.1374	0.1311	0.0586			
FNV-Yale	0.1016	0.0949	0.0646	0.0320	0.0214	0.0700
FNV-IP	0.1032	0.0992	0.0602	0.0305	0.0200	0.0713
FNV-FC	0.1017	0.0967	0.0639	0.0308	0.0202	0.0682
FNV-NT	0.1036	0.1000	0.0595	0.0348	0.0199	0.0934
Asibi	0.1035	0.0975	0.0636			
17D-204	0.1056	0.1017	0.0582	0.0290	0.0190	0.0693
**B**	**Consensus FNV Genotype (n** = **26)**					
FVV	0.1266	0.1303	0.0650			
FNV-Yale	0.0184	0.0143	0.0262	0.8829	0.8849	0.0091
FNV-IP	0.0242	0.0202	0.0285	0.8815	0.8848	0.0097
FNV-FC	0.0703	0.0205	0.0897	0.8599	0.8798	0.0465
FNV-NT	0.0700	0.0454	0.0680	0.6498	0.8744	0.3805
Asibi	0.1003	0.0941	0.0748			
17D-204	0.0946	0.0906	0.0680	0.1266	0.0241	0.2808
**C**	**Main Revertants (n** = **8)**					
FVV	0.1203	0.1169	0.0693			
FNV-Yale	0.0355	0.0202	0.0429	0.8857	0.8849	0.0047
FNV-IP	0.0387	0.0275	0.0455	0.8849	0.8848	0.0054
FNV-FC	0.1482	0.1377	0.0433	0.8397	0.8400	0.0328
FNV-NT	0.1165	0.0986	0.0910	0.4661	0.4998	0.4416
Asibi	0.1287	0.1141	0.0978	0.1012	0.0192	0.2079
17D-204	0.1093	0.0906	0.0863	0.0337	0.0172	0.0416

(**A**) NSE and RMSD along the entire open reading frame of the virus. (**B**) NSE and RMSD along the 26 nucleotide positions containing the putative FNV genotype. (**C**) NSE and RMSD for the eight nucleotide positions containing overt amino acid residue instability and reversion to wild-type identity. Wild-type viruses Asibi and FVV are more diverse than their related vaccine strains, and more neurotropic FNV strains are of less diversity than the others; the pattern is especially visible at narrower scales.

#### For 26 Sites Composing the Putative FNV Genotype

NSE was calculated along the 26 nucleotide positions encoding the putative FNV genotype (Table 2), and is shown in Table 3A and Fig. 1E. A single-factor Kruskal-Wallis test showed a significant difference for NSE within the aggregate group (X^2^ = 67.348, df = 6, p < 0.001). A pairwise Wilcoxon sign-rank test revealed significant differences in NSE between all pairs considered (n = 7), except for the pairs Asibi/FVV (p = 0.11), Asibi/FNV-NT (p = 0.06), Asibi/17D-204 (p = 0.75), FVV/17D-204 (p = 0.06), FNV-FC/FNV-IP (p = 0.10), FNV-FC/FNV-NT (p = 0.53), FNV-Yale/FNV-IP (p = 0.16), and FNV-NT/17D-204 (p = 0.11). Likewise, RMSD was calculated along the 26 positions, and is shown in Table 2B and Fig. 1F. A single-factor Kruskal-Wallis test showed a significant difference of RMSD between the strains at these sites (X^2^ = 131.87, df = 6, p < 0.001).

#### Diversity Loss for 17D-204 from Asibi at Known Genotype

NSE of Asibi and 17D-204 was compared using the 20 amino acid substitution sites defined by international standards to be encoded by all substrains of 17D-204. NSE of Asibi at these sites (mean = 0.118, median = 0.133, s.d. = 0.0738) was greater than that of 17D-204 (mean = 0.029, median = 0.015, s.d. = 0.046), and this difference was statistically significant by Wilcoxon test (*W* = 361, p < 0.001) (Fig. 1B). The loss of diversity at these sites is expected, and consistent with previous reports^15^.

#### Error Rates for Open Reading Frames

**Figure 2:**
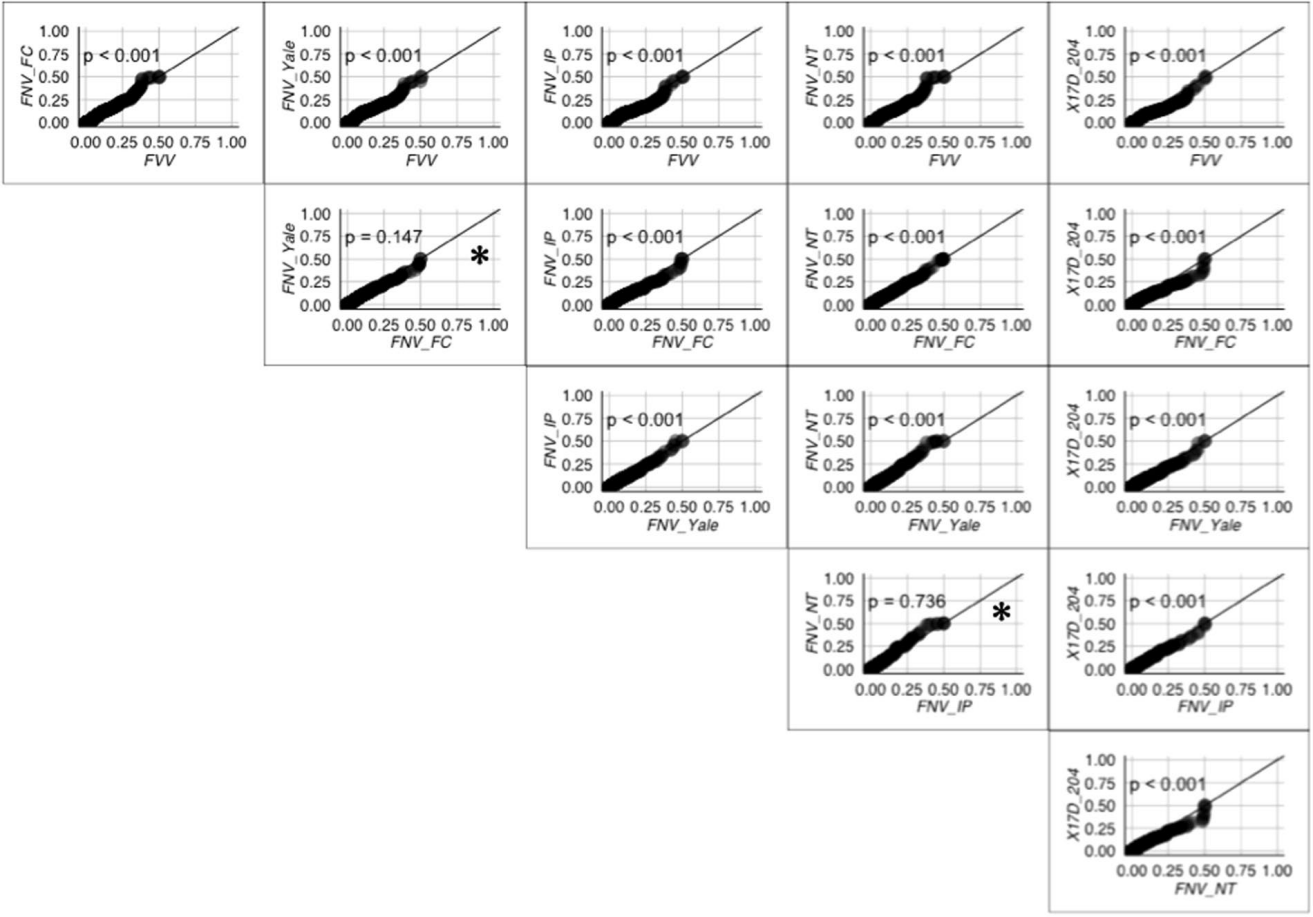
Pairwise quantile-quantile plots of sequencing error rate for FVV and FNV strains. In this case, error rate contains both true variant populations and sequencing artifacts. The presence of non-consensus variant frequencies is observed by aggregation of points away from a slope of 1. P-values were generated by a bootstrapped Kolmogorov-Smirnov test, using a Bonferroni-corrected alpha of 0.008. Under these criteria, all pairings differ significantly except those of FNV-NT/FNV-Yale and FNV-NT/FNV-IP (*non-significant); this supports other observations in the study that FNV-NT and either FNV-Yale/IP are highly divergent in their subpopulations.

Error rates for the alignments were compared using quantile-quantile plots and bootstrapped Kolmogorov-Smirnov (KS) tests; this procedure revealed significant divergence between the distribution of error rate for all pairings of strains, except between pairs FNV-IP/FNV-NT and FNV-Yale/FNV-FC (Fig. 2).

#### Analysis of Variant Structure

**Figure 3:**
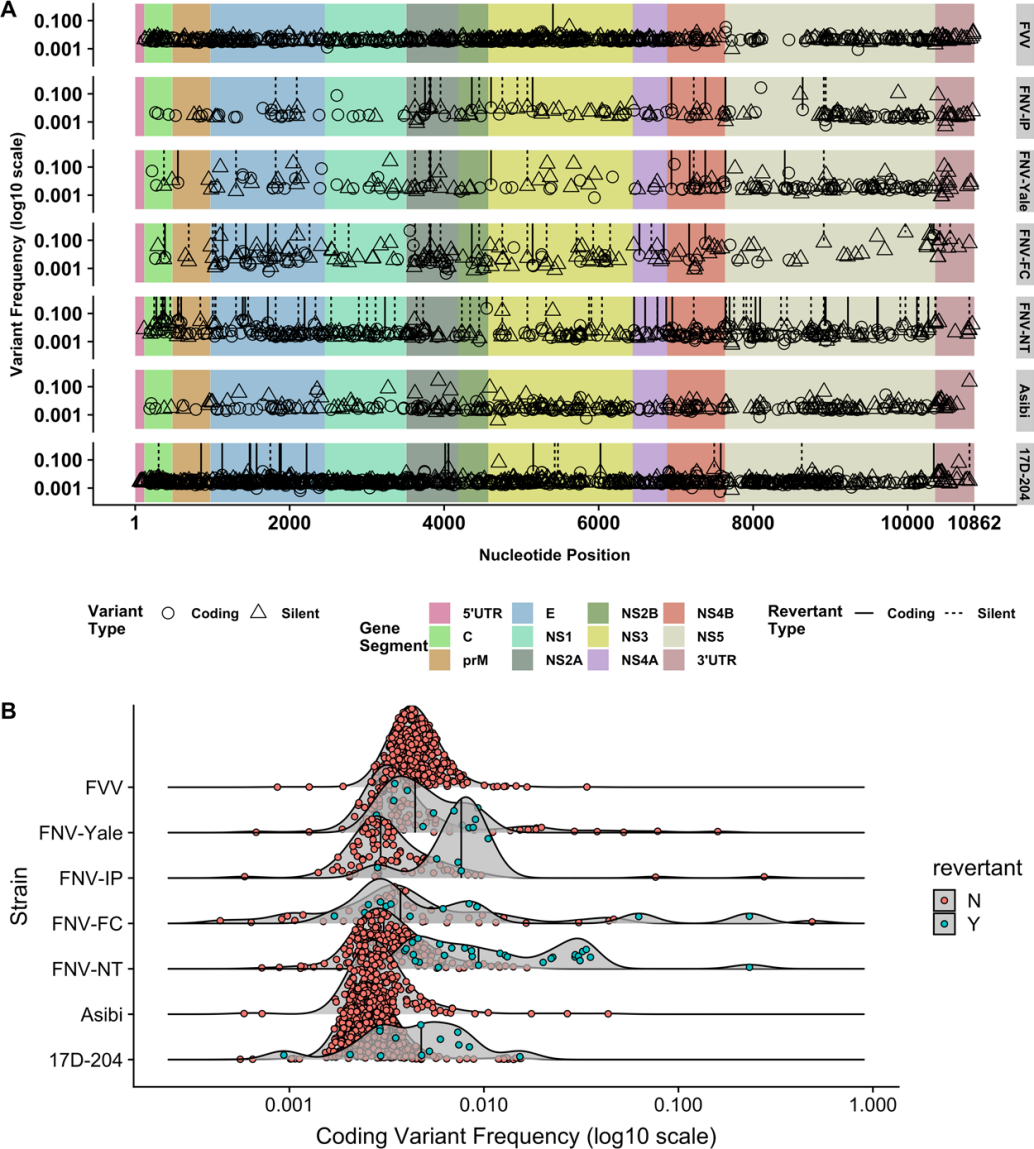
Summary of single-nucleotide polymorphism (SNP) content for YFV strains considered in the study. **(A)** SNPs were modeled using V-Phaser v.2.0; significantly represented variants were mapped to the consensus sequence of the virus from which they were detected. Variants are depicted along the YFV genome with respect to their frequency in the alignment, with gene boundaries shown; those generating an amino acid substitution are shown by symbol. SNPs encoding reversion to the wild-type (FVV or Asibi for 17D-204) genome are shown as vertical lines. **(B)** Plots of density for detected variants that code for amino acid substitutions, separated by wild-type revertant identity. Viruses with homogenous population structure (17D-204, FNV-Yale, FNV-IP) cluster at the lower end of the frequency range, whereas the less homogenous viruses (FNV-NT, FNV-FC) show density of counts at relatively higher ranges, with greater relative density of high-frequency revertants. Median count value is depicted by a vertical line.

Subpopulation, single-nucleotide variants were recovered in all of the read alignments analyzed in the study, with differences observed between the vaccine strains for raw counts of variants and overall distribution of the frequency of the variants. The results of this test support the observation by consensus sequence that a number of nucleotide positions in the read alignments encode reversions to wild-type identity, as defined by the FVV consensus sequence used here (Fig. 3A,B). For the highly neurotropic strains FNV-Yale and FNV-IP, the numbers of observed variants was equivalent (n = 91, both strains); for the low-neurotropism strains FNV-NT and FNV-FC, a greater abundance of variants was recovered from the read alignments, and these were at greater frequency that the high-neurotropism strains (Table 4). Vaccine strains (FNV, 17D) contained fewer variants than their parental strains (FVV, Asibi).

**Table 4:** Descriptive statistics for single-nucleotide variants observed in the read alignments of each virus.

	Total Coding Variants				Revertants to Wild-Type			
	Frequency				Frequency			
	n	Mean	Median	s.d.	n	Mean	Median	s.d.
FVV	253	0.0049	0.0045	0.0024				
FNV-Yale	91	0.0072	0.0033	0.0164	13	0.0049	0.0047	0.0016
FNV-IP	91	0.0191	0.0043	0.0441	13	0.0077	0.0090	0.0033
FNV-FC	131	0.0272	0.0074	0.0533	35	0.0421	0.0055	0.0871
FNV-NT	165	0.0086	0.0040	0.0178	52	0.0122	0.0080	0.0086
Asibi	154	0.0103	0.0034	0.0299				
17D-204	351	0.0037	0.0026	0.0052	8	0.0091	0.0046	0.0091

Although raw counts of polymorphic sites are irregular, the frequencies of these SNPs are lower for the vaccine strains than for the parental viruses; frequencies of SNPs are higher for FNV strains with low neurotropism (FNV-FC, FNV-NT).

#### Overt Reversion and Instability

Eight nucleotide positions were observed to be especially unstable between the FNV strains, showing both high diversity and high genetic distance from FVV for at least one of the FNV strains sequenced (Fig. 1E,F). These were, respectively, 1572 (E-T200K), 4607 (NS3-I13V), 6942 (NS4B-L19S), 7178 (NS4B-V97I), 7380 (NS4B-A165V), 7641 (NS5-2RT), 8409 (NS5-I258T), 8640 (NS5-R335K) (Table 3C). For these positions, the presence of low-frequency revertants was most evident in highly neurotropic strains FNV Yale and FNV-IP; these reversion events in some cases proceed to consensus levels in the low-neurotropism strains FNV-FC and FNV-NT (Table 2), and so were not detected by the model when the revertant nucleotide was of majority identity (Table 5).

**Table 5:** For the nucleotide sites containing overt reversion to wild-type amino acid identity, the individual percentage of the revertant amino acid are depicted.

Substition	Strain				
	FNV-Yale	FNV-IP	FNV-FC	FNV-NT	17D-204
1572 (E-T200K)	—	—	—	—	0.0936
4607 (NS3-I13V)	1.0520	0.5503	—	*	—
6942 (NS4B-L19S)	0.7647	0.2803	—	*	—
7178 (NS4B-V97I)	—	0.3481	—	—	—
7380 (NS4B-A165V)	0.8807	0.9309	6.2540	*	—
7641 (NS5-2RT)	0.2839	0.4039	—	*	—
8409 (NS5-I258T)	—	0.3439	—	—	—
8640 (NS5-R335K)	0.7361	—	—	—	—

There is concordance of these variants for FNV-IP and FNV-Yale, while the SNPs are not observed in FNV-FC and FNV-NT. *Reversion to wild-type by consensus; would not be detected by the variant scan.

## Discussion

Previous to the molecular era and viral quasispecies paradigm, population diversity had been hypothesized to influence the properties of YFV vaccines. Some baseline of population diversity has been observed in YFV vaccine strains, for which evidence includes the recovery by plaque purification of not only rare clonal neurotropic variants, but also the detection of low-level antigenic variants of 17D by monoclonal immunoassay^19,20^. In 1935, the presence of a selectable population of virus alleles was proposed to explain the rapid adaptation of FNV to serial intrahepatic passage in rhesus macaques, in which the vaccine strain gained a wild-type viscerotropic phenotype^21^. Efficient phenotype selection was also shown for 17D, for a study in which, monkey neurotropism was efficiently rescued by passage of the 17D vaccine strain in mouse brain, showing increased morbidity after a single passage cycle^22^. These studies and others suggest a paradigm by which adverse variability YF vaccines may result from poorly controlled empiric adaptation.

We have previously reported a massively parallel (deep sequencing) approach to analyze population structure of the commercial YFV vaccine strain 17D-204 (YF-Vax®), showing that the vaccine was of lower diversity than the wild-type parental strain Asibi^15^. From these previous results, it was postulated that low population diversity would contribute to the considerable safety record of 17D vaccine derivatives. The current study confirmed the previous results for Asibi and 17D (YF-Vax®), in which the vaccine was observed to be of significantly lower diversity than the parental strain. This pattern is recapitulated for comparisons of FVV and FNV. Furthermore, the pattern is overt for the 26 nucleotide substitutions form the putative consensus genotype for FNV.

While the general pattern of low diversity is repeated here for FNV, there is considerable population diversity that is not shared between the vaccine strains. This supports previous data that suggests that certain collection strains of FNV may not have been stably fixed. Unfortunately, the relationship of the four FNVs in this study to the original “Dakar” strain of FNV is not known. Historical documentation attests that FNV lots were produced from seeds of restricted passage level, controlling for sterility, potency, and immunogenicity in monkeys^6^. Laboratory passage histories are not available for the strains that were handled in this study from any of the source institutions, however, the previously observed differences in mouse and monkey neurovirulence for these strains provides a framework by which the RNA population structure of the viruses may be associated with the adverse phenotype of the vaccine. A comparison of FNV neurovirulence in mice showed that FNV-Yale produced a shorter average survival time (AST) following both intracerebral and intranasal inoculation when compared to FNV-IP and FNV-NT, while FNV-FC had an extended AST compared to the three other strains^17^. Likewise the FNV-NT strain, observed in this study to be the most divergent of FNV vaccines, was previously recognized by Wang *et al*. to be nonlethal in cynomolgus monkeys (AST >30 days) whereas FNV-IP and FNV-FC were lethal^17^. Thus, FNV-Yale has the mouse and monkey neurovirulence properties described for the original “Dakar” FNV strain of the vaccine. It is plausible that our observed low abundance of variant amino acid substitutions in FNV-Yale compared to the three other FNV strains, both by consensus sequence and by variant reconstruction, reflects a short and protected passage history from the original vaccine.

Relative to FVV, we observed conservation of 19 amino acid substitutions for the four FNV examples sequenced (C (2), prM (1), M (1), E (4), NS2A (2), NS2B (1), NS3 (1), NS4A (2), NS4B (2) and NS5 (3)) (Table 2), suggesting that these are the particular amino acids involved in the derivation of FNV from FVV; however, we cannot exclude that other mutations in the original FNV have been lost on passage of the four FNVs studied here. For example, several amino acid substitutions are conserved across three of the vaccines, but not in the most divergent strain, FNV-NT, which significantly had lost the monkey neurovirulence phenotype (Table 1). By RMSD, the genetic distance from parental consensus identity is bifurcated (Table 3); a number of sites harbor particularly overt reversion to wild-type identity. This finding suggests that these particular mutations in the vaccine are selected to revert under laboratory conditions, and that maintenance of this small set of mutations (n = 8) may influence the neurotropic phenotype of FNV (Table 2).

Notably, the substitutions M-L36F, E-K200T, E-K331R, and NS4B-I95M were observed in common for the attenuation processes between FVV to FNV, and Asibi to 17D (Table 2). Flavivirus M protein contains a pro-apoptotic domain that, upon transfection and overexpression, is attenuated in phenotype when phenylalanine is substituted at residue 36^23^. Since the substitution at E-K200T is shared by Asibi and all FNV strains, it is likely either a cell culture adaptation or compensatory, and not consequential to the attenuated phenotype. Despite being associated with mouse neuroinvasion the residue did not singularly affect the phenotype of a cloned virus^24^. The residue E-331R was associated with both attenuation of Asibi to yield 17D and experimental adaptation of the Asibi strain to hamster liver, so the contribution to vaccine attenuation is complicated by the paucity of animal models and the difficulty to parse neurotropic from viscerotropic determinants of pathogenicity^25^. The function of NS4B mutations in attenuation of the vaccine is not understood, however, evidence has been put forth to suggest roles for NS4B in both interferon antagonism and replication complex associations^26,27^.

Reversion to wild-type is observed for FNV strains at not only consensus, but also for small population variants. For raw variant counts and revertants, the strains varied in their divergence from the original vaccine; e.g. FNV-IP and FNV-Yale alignments revealed the relatively low counts of both variants and revertants. Conversely, counts of variants and revertants were higher in FNV-FC and FNV-NT, which are hypothesized to be the most phenotypically divergent from the original FNV strain. Though genome-scale estimations of diversity are similar between all FNV strains and 17D-204; as expected, diversity of FVV was greater than for the vaccines. Processing and alignment methods used in these analyses were more stringent than previously used for the Asibi strain, reducing observable differences in estimated diversity between Asibi and 17D-204^15^. However, density comparison shows both that 17D-204 variants are fixed to lower aggregate frequencies than those of Asibi, FVV, or the FNV vaccines, and that the wild-type strains contain a population of higher frequencies for coding variants (Fig. 3A,B). This pattern may contribute to the superior safety record of 17D vaccine compared to FNV, and supports a hypothesis that diversity influencing the vaccine phenotype arises from a limited number of sites, and that some level of low-frequency variants are tolerated in fixed vaccine preparations.

An alignment of reference sequences for Asibi [gbAY640589.1] and FVV [gbYFU21056.1] shows 21 nucleotide differences, of which 8 are coding changes (not shown). The presented study indicates lesser divergence of the parental strains than was previously described by Sanger methods undertaken 20 years ago, revealing instead only 11 consensus nucleotide differences and two amino acid substitutions^17^. FVV and Asibi were both isolated in 1927 at considerable geographic separation (Senegal and Ghana, respectively), however the limited divergence observed in this study supports a conclusion that both parental strains originated from the same YF epidemic.

## Methods

### Viruses

Wild-type French viscerotropic (FVV) and Asibi strains were obtained from the World Reference Center for Emerging Viruses and Arboviruses (Galveston, TX, USA) as lyophilized cell culture supernatant. “FNV-IP” was obtained from the Institut Pasteur, (Paris, France). “FNV-Yale” was obtained from the Yale Arbovirus Collection, (New Haven, CT, USA). “FNV-FC” was obtained from the Centers for Disease Control and Prevention (Fort Collins, CO). “FNV-NT” was obtained from what is now called Public Health England, Porton Down (Salisbury, UK)^12^. Vaccine strain 17D-204 was obtained from a commercial ampoule of YF-Vax® lot UH356AA (Sanofi-Pasteur, Swiftwater, PA) (Table 1). Asibi and 17D-204 were included in the study as method controls; they were previously analyzed for their diversity profiles and provide an expected baseline of the comparative relationship of vaccine to wild-type parental strain^15^. The passage history of all four FNVs from originating FNV vaccine is unknown; their *in vivo* phenotypic properties have been described in mouse and monkey and are cited in Table 1^17^. All vaccine strains were passaged once only in Vero cells in this study to produce working stocks using Eagle’s minimal essential media, supplemented with 2% fetal bovine serum, L-glutamine, and non-essential amino acids (5% CO_2_, 37 °C). Wild-type strains FVV and Asibi were handled at BSL-3 containment, vaccine strains were handled at BSL-2.

### RT-PCR Amplification Strategy

Wild-type FVV and Asibi strains were reconstituted in sterile, molecular-grade water. Vaccine strain 17D-204 was reconstituted using the provided injection diluent. RNA was isolated from reconstituted seed stocks of FVV and all working stocks of FNV by column isolation using the QiAmp kit (Qiagen, Gaithersburg, MD), and amplified by RT-PCR to produce six overlapping amplicons as previously described^15^.

### Sequencing and Data Preparation

Pooled libraries were prepared using Nextera DNA kits (Illumina), and sequenced on an Illumina Hi-Seq-1500 instrument to yield 50b, paired-end reads. Reads were trimmed for primer sequences using Trimmomatic v, 0.30, and filtered to retain reads with mean quality scores above 35 (Illumina v.1.9 encoding). *De novo* assembly of reads was performed with ABySS v.1.5.2, using the paired-end function and kmer sizes from 20–40. Bowtie v. 2.1.0 was used to locally realign all read sets to the publicly available sequence of FVV [gbYFU21056] using “very sensitive” presets. PCR duplicates were removed from alignments using Picard Tools MarkDuplicates v.1.107. Alignments were randomly downsampled to normalize read counts using Picard Tools DownsampleSam v.1.107. File conversion, inspection, and compression tasks were performed using Samtools with HTSlib v.1.0. Downsampled coverage statistics are shown in Table 1 and Fig. 1A.

### Generation and Comparison of Consensus Sequences

Consensus sequences were prepared for all strain alignments using bcftools v.1.0, using the biallelic variant caller with haploid settings; conversion of sequences to FASTA was performed with vcfutils.pl and GNU awk v.4.1.0. Resulting sequences were aligned to wild-type FVV using Macvector v.13.0.7, and compared for divergence from the parental strain. Reads from previously reported sequencing of the Asibi strain (wild-type parent of the 17D strain) and 17D-204 vaccine were reprocessed identically to FNV strains; consensus sequences from FVV and Asibi were similarly aligned and compared^28,29^.

### Comparison of Diversity And Genetic Distances

Read alignments were analyzed for the population diversity of viral RNAs, which is quantitated by index measurements that derive from the relative frequencies of nucleotide identities that align to a position in the reference sequence. For each virus sequenced, nucleotide counts for all strain alignments were parsed from BAM files using the R library deepSNV v.1.8.0, and relative frequencies were computed for the possible nucleotide set {*A*, *C*, *G*, *U*, −}, which includes a gap character that may be introduced by the alignment algorithm. Error rate *ER* of the alignment *X* at nucleotide position *i* is defined as the remaining nucleotide frequencies after subtracting the frequency of the maximum value of the set, the frequency of the presumed consensus nucleotide.

$$ER{(X)}_{i}=1-max\{A,C,G,U,-\}$$

In the same manner, diversity of nucleotides at each position *i* were estimated using relative frequencies normalized Shannon’s entropy *NSE* and normalized to 1.61, the maximum value of the index. The probability *P* refers to the frequency of any nucleotide in the set *p*, which are log-transformed and summed.

$$NSE{(X)}_{i}=-\,\sum _{p=\{A,T,C,G,-\}}\,P[{X}_{i,p}]ln(P[{X}_{i,p}\mathrm{])/1.61}$$

Additionally, genetic distance from wild-type was estimated by root-mean square distance *RMSD* at each nucleotide position in two read alignments *X* (wild-type) and *Y* (vaccine). The squared difference in nucleotide frequency between alignments *X* and *Y* is obtained, squared, summed, and normalized. *RMSD* values for a nucleotide position may range from 0 (no change) to 0.632 (complete selection of an alternate consensus nucleotide.

$$RMSD{(X,Y)}_{i}=\sqrt{\frac{{\sum }_{p=\{A,T,C,G,-\}}{(P[{X}_{i,p}]-P[{Y}_{i,p}])}^{2}}{5}}$$

The simultaneous use of both diversity and distance measurements is necessitated by the possibility that nucleotide diversity may be of both low diversity and high genetic distance from the reference alignment; this would be a case of complete selection of an alternate consensus nucleotide. Changes in diversity and relative genetic distance measurements were estimated along scales of (1) the single open reading frame of the virus and (2) sets of nucleotide positions which define the consensus genotype differences between wild-type and vaccine. For this study, the “FNV” nucleotide set consists of the 26 positions encoding amino acid substitutions in at least 3 of 4 FNV strains, relative to the parental strain FVV. The “17D” nucleotide set consists of the 20 positions encoding amino acid substitutions in 17D strain vaccines, relative to the parental Asibi strain^14^.

### Analysis of Variant Structure

For each alignment, variants were modeled using V-Phaser v.2.0, with default settings and a significance cutoff of 0.05^30^. Variants were classified against the consensus codon as silent or coding using SNPdat.pl^31^, and then were inspected for reversion to wild-type identity. Identified variant sets were inspected for the presence of coding polymorphisms that arose from nucleotide positions with large shifts in diversity between the strains, under a hypothesis that these would be most likely to influence previously identified differences of *in vivo* neurotropism.

### Statistics

Error rate distributions were compared for each pair of alignments using quantile-quantile plots, followed by bootstrapped Kolmogorov-Smirnov tests. NSE and RMSD were compared nonparametrically across the scale noted using single-factor Kruskal-Wallis followed by pairwise Wilcoxon signed-rank tests, using an alpha of 0.05 and bonferroni correction. Variants were modeled from paired-end Illumina read alignments using the method of Yang^30^, using default false discovery rate settings. All statistical tests were performed in base R v.3.4.4, with graphs generated using ggplot2.

### Accession codes

Consensus sequences for strains sequenced in this study were submitted to genbank under the following accession codes: MH444798 (FVV), MH444794 (FNV-FC), MH444795 (FNV-IP), MH444796 (FNV-NT), MH444797 (FNV-Yale). Illumina read sets were submitted to SRA under the following NCBI BioSample accession codes: SAMN09302786 (FVV), SAMN09302787 (FNV-FC), SAMN09302788 (FNV-IP), SAMN09302789 (FNV-NT), SAMN09302790 (FNV-Yale).
